# Frontline staff motivation levels and health care quality in rural and urban primary health facilities: a baseline study in the Greater Accra and Western regions of Ghana

**DOI:** 10.1186/s13561-016-0112-8

**Published:** 2016-08-31

**Authors:** Robert Kaba Alhassan, Edward Nketiah-Amponsah

**Affiliations:** 1 Amsterdam Institute for Global Health and Development, University of Amsterdam, Amsterdam, Netherlands; 2 Department of Epidemiology, Noguchi Memorial Institute for Medical Research, University of Ghana, Legon, Ghana; 3 Department of Economics, University of Ghana, Legon, Ghana

**Keywords:** Ghana, Rural–urban, Health worker motivation, Quality health care, Health facilities

## Abstract

**Background:**

The population of Ghana is increasingly becoming urbanized with about 70 % of the estimated 26.9 million people living in urban and peri-urban areas. Nonetheless, eight out of the ten regions in Ghana remain predominantly rural where only 32.1 % of the national health sector workforce works. Doctor-patient ratio in a predominantly rural region is about 1:18,257 compared to 1:4,099 in an urban region. These rural–urban inequities significantly account for the inability of Ghana to attain the health related Millennium Development Goals (MDGs) before the end of 2015.

**Purpose:**

To ascertain whether or not rural-urban differences exist in health worker motivation levels and quality of health care in health facilities accredited by the National Health Insurance Authority in Ghana.

**Methods:**

This is a baseline quantitative study conducted in 2012 among 324 health workers in 64 accredited clinics located in 9 rural and 7 urban districts in Ghana. Ordered logistic regression was performed to determine the relationship between facility geographic location (rural/urban) and staff motivation levels, and quality health care standards.

**Results:**

Quality health care and patient safety standards were averagely low in the sampled health facilities. Even though health workers in rural facilities were more de-motivated by poor availability of resources and drugs than their counterparts in urban facilities (*p* < 0.05), quality of health care and patient safety standards were relatively better in rural facilities.

**Conclusion:**

For Ghana to attain the newly formulated sustainable development goals on health, there is the need for health authorities to address the existing rural–urban imbalances in health worker motivation and quality health care standards in primary healthcare facilities. Future studies should compare staff motivation levels and quality standards in accredited and non-accredited health facilities since the current study was limited to health facilities accredited by the National Health Insurance Authority.

## Background

In Ghana, the number of urban dwellers has increased because of unabated rural-urban migration. According to the Ghana Statistical Service (GSS), as at 2015 approximately 70 % of the estimated 26.9 million population lived in urban and peri-urban areas. Nonetheless, eight out of the ten regions in Ghana remain predominantly rural where only 32.1 % of the national health sector workforce works [1].

Over the years, equitable access to good quality healthcare has been a national challenge for many developing countries including Ghana. The percentage of deliveries attended to by skilled health workers in 2010 in the Northern region (one of the poorest and largely rural regions) was 36.8 % compared to 54.4 % in the Greater Accra region (which is largely urbanized). Likewise, the doctor-patient ratio in 2010 was about 1:4,099 in the Greater Accra region compared to 1:18,257 in the Northern region [2].

Thirty percent (30 %) of the estimated population of 11,579 doctors and nurses work in rural areas while the remaining 70 % work in urban areas. In addition, physician density per 1000 population in urban Ghana is estimated to be 0.13 compared to 0.04 in rural areas while that for nurses is 0.60 per 1000 population in urban areas compared to 0.20 in rural areas [3]. Physician assistants and midwives are the only cadre of professionals mostly in rural areas in Ghana. Out of the total population of 712 physician assistants and 4,929 midwives, 70 and 60 % of them respectively work in rural areas because these cadres of health professionals are posted to work in primary level health facilities which are often in rural areas [3].

A number of factors have been cited as contributing to the desire of Ghanaians, including health workers, to live and work in urban areas. These factors include inadequate social amenities and limited opportunities for career and educational development in rural areas [3]. Understaffing of health facilities and inadequate health infrastructure, especially in rural areas, have created wide inequities in access to good quality health care and the inability to attain all the health related Millennium Development Goals (MDGs) [2, 4–6].

The Government of Ghana (GoG) through the Ministry of Health (MoH) has been implementing a number of interventions to ensure equitable distribution of health sector human resources. Some of these interventions include payment of rural allowance of up to 30 % of monthly salary to health staff who accept posting to rural areas, offering post basic education courses and vehicles on hired purchased basis [3], even though full implementation of these policies remains a challenge.

Notwithstanding these interventions, the rural–urban disparities in human resource distribution and quality health care delivery persist, raising concerns on the effectiveness of these interventions in motivating healthcare workers to accept posting to deprived areas [6, 7].

Apart from these health worker motivation interventions, the National Health Insurance Scheme (NHIS) was implemented in 2005, as a social intervention policy, to ensure financial protection and risk pooling for Ghanaians, especially the poor in rural and urban areas. Under the NHIS, indigents, pregnant women, people aged 70 years and above, and children under 18 years are exempted from premium payments. These categories of people, put together, constitute 63.1 % of the total NHIS subscriber base [8].

To sustain the NHIS and guarantee scheme subscribers of continuous universal access to good quality care there is the need to maintain quality care standards in accredited health facilities and ascertain the workplace incentives and capacity challenges for staff working in these health facilities.

Previous studies on health worker motivation in Ghana [6, 7, 9, 10] did not examine the rural–urban differences in quality of health care delivery and staff motivation levels in NHIS accredited health facilities. This study thus sought to ascertain the rural–urban differences in staff motivation levels and quality health care standards in 64 NHIS-accredited primary health care facilities.

It is expected findings of this study will contribute to existing knowledge and debate on rural and urban dynamics in population health in Ghana. Findings of this study are also expected to inform policy discussions on rural–urban mainstreaming in health sector human and material resources allocation and distribution.

## Methods

### Study design and sampling strategy

This is a semi-structured baseline study conducted in 2012 in the Greater Accra and Western regions of Ghana. These two regions were purposively sampled to avoid spill-over effect since they do not share a common boundary. This condition was needed to ensure effective implementation of planned interventions which form part of a broader WOTRO- COHEiSION project^1^ out of which this paper is written. Moreover, these two regions were selected because of financial and time constraints which limited selection of regions in the northern part of Ghana. The study was conducted in 38 private and 26 public health care facilities in 9 rural^2^ and 7 urban^3^ districts.

Principal Component Analysis (PCA) was used to select the study districts in the two regions based on district similarities in (1) number of NHIS accredited facilities, (2) population size, and (3) NHIS client enrolment rate. Eight (8) districts with closest PCA scores were selected from each region to ensure they were homogeneous and comparable in several respect. This approach was meant to help measure the impact of implemented interventions in subsequent follow-up surveys in 2014.

At the health facility level, the PCA was used to generate scores for NHIS accredited primary healthcare facilities in the Greater Accra Region (GAR) and Western Region (WR). Variables used for the PCA were facility ownership, location and accreditation scores on: range of services; staffing; environment and infrastructure; basic equipment; organization and management; safety and quality management, and outpatient services.

Using the quota system, each selected district in a region was allocated a maximum of 4 qualified facilities. Per this criterion, a total of 32 public and private facilities were randomly sampled from each region irrespective of region size and number of health facilities in these regions. This strategy ensured that all selected 64 facilities were comparable in several respects.

**Fig. 1:**
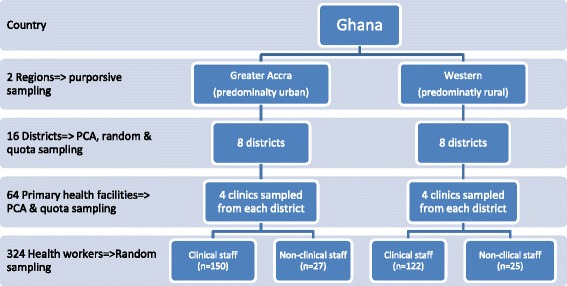
Sampling strategy

At the staff level, clinical and non-clinical staff^4^ were randomly sampled and interviewed from all 64 facilities. At least six (6) health workers were earmarked for random selection and interview in each clinic. As shown in Fig. 1, in both Greater Accra and Western regions, clinical staff (*n* = 272) dominated non-clinical staff (*n* = 52).

All available workers were interviewed in clinics that had total staff strength less than six (6). Inclusion criteria for staff were full time employment and at least 6 months work experience. This strategy was used to elicit responses from staff who knew much about their work environment. In all, 333 questionnaires were administered to respondents out of which 324 were correctly filled representing a 97 % return rate.

### Data collection instruments

Data collection instruments used were a nineteen (19) paged clinic staff questionnaire and a clinic quality health care assessment tool called *SafeCare Essentials*. The tool is provided by the SafeCare Initiative, a collaboration of PharmAccess Foundation, Council for Health Services Accreditation of Southern Africa (COHSASA), and Joint Commission International (JCI). *SafeCare Essentials* was developed based on JCI’s International Essentials of Health Care Quality and Patient Safety.

The quality health care assessment was based on five (5) main components, namely: (1) leadership processes and accountability, (2) competent and capable workforce, (3) safe environment for staff and patients, (4) clinical care of patients and (5) improvement of safety and quality. Forty-one (41) questions were asked under the five (5) components on four levels of effort (0–3) with low levels of effort depicting low performance and *vice versa*.

Zero (0) is scored when the desired quality improvement activity in a clinic is absent, or there is mostly ad hoc activity related to risk reduction. One (1) is scored when more uniform risk-reduction activity begins to emerge in a clinic. Two (2) is scored when there are processes in place for consistent and effective risk-reduction. Three (3) is scored when there is data to confirm successful risk-reduction strategies and continuous improvement.

To avoid bias during administration of the *SafeCare Essentials* tool, double scoring was done by three trained research assistants using Pocket Digital Assistant (PDA) devices. As part of the assessment process, clinic administrative records were reviewed alongside observations and key informants’ interviews. For the purposes of this analysis, the four levels of effort towards patient safety and quality health care in pertinent facilities were dichotomized into two by combining levels 0 & 1 into “low level of effort” and levels 2&3 into “high level of effort”.

Staff motivation was measured using proxies such as physical work conditions, monthly salary, possibility for promotion/further education, and recognition gained from job. Nineteen (19) questions were asked on workplace motivation factors. Rating on these factors were done on a four-point Likert scale from 1 = “very disappointing” to 4 = “very satisfactory”.

Piloting of data collection instruments was done in two conveniently sampled clinics in Greater Accra region to correct typographical mistakes and ensure conversance with the questions and the interview process.

### Data management and analysis

The data set was split into rural and urban sub- samples for comparative analysis on rural–urban differences in the various outcome variables of interest. All data sets were analyzed using the STATA statistical software (version 12.0). Parametric (ordered logistic regression) and non-parametric (Pearson Chi-square, Wilkoxon Mann–Whitney rank sum) tests were conducted to test the hypothesis that health worker motivation and quality health care in rural and urban health facilities are not different.

In addition, factor analysis was conducted with orthogonal varimax rotation (Kaiser off) to group the 19 workplace motivational factors into four major factors [11]. Based on Bennette and Franco’s [12 ceptual framework, these four factors were predicted and named as follows: (1) clinic physical work environment, (2) resource and drugs availability, (3) financial and extrinsic incentives, (4) job prospects and career development. Cronbach’s alpha (α) was conducted to check for scale reliability of the 19 Likert scale items and found to be 0.82 which was above the 0.70 rule of thumb [13].

Summary and descriptive statistics were also performed on staff socio-demographics and situational analysis of health facilities. Differences in personal experiences of health workers in rural and urban health care facilities were analysed based on transportation to work, tardiness to work, extra work hours, workload, moonlighting and overall rated satisfaction with work incentives.

Variables that assessed satisfaction levels with work conditions by health personnel on a four-point Likert scale from 1 = “very disappointing” to 4 = “very satisfactory” were dichotomized into two by combining 1 & 2 into “disappointing” and 3 & 4 into “satisfactory”. This allowed for easy presentation and interpretation of results. Situational analysis was also done in the 64 sampled health facilities on available staff, material resources and health care services delivered per month. Situational analysis, as used in this context, entails the instant assessment of health facility physical condition and service delivery processes on the day of visit.

Differences in quality health care standards in rural and urban facilities were explored using the *SafeCare Essentials* risk assessment tool which has also been used in Alhassan et al. [14].

## Results

### Characteristics of health workers interviewed

The results showed that respondents working in rural health facilities 182 (56 %) dominated those in urban health facilities 142 (44 %). In terms of gender distribution, female health workers 217 (67 %) dominated male workers 107 (33 %) in both rural and urban settings. Majority of the respondents (59 %) in rural and urban facilities aged 40 years or below; 29 % aged between 41 and 60 years and 12 % were 61 years and older. Averagely, health workers in urban facilities were older (mean = 42 years) than their counterparts in rural facilities (mean = 36 years, *p* = 0.0010). The mean age of respondents in rural and urban health facilities was 39 years.

In terms of educational qualifications of respondents, close to 50 % had a least tertiary education; 34 % had secondary education and 20 % did not indicate their educational qualification. Clinical staff constituted the majority of respondents representing 84 % compared to 16 % of non-clinical staff.

As shown in Table 1 close to 60 % of the health workers said they receive monthly salary equivalent to US$ 265 or less; 39 % receive between US$ 265- US$ 688 and 1 % receive more than US$ 688 as monthly salary; 22 % of the married staff were from urban facilities while 35 % of unmarried staff worked in rural facilities (*p* = 0.046). Overall, 43 % of the respondents were married while the remaining 57 % were unmarried. Christianity was mentioned by 96 % of the staff as their religion while 4 % of the respondents mentioned other forms of religions (See Table 1).

**Table 1:** Characteristics of health staff (*n* = 324)

	Rural	Urban	Total	
Variables	Freq. (%^b^)	Freq. (%)	Freq. (%)	*p*-value
Gender				0.354
Male	64 (20)	43 (13)	107 (33)	
Female	118 (36)	99 (31)	217 (67)	
Age				0.204
≤ 40 years	118 (36)	73 (23)	191 (59)	
41–60 years	44 (14)	49 (15)	93 (29)	
≥ 61 years	20 (6)	20 (6)	40 (12)	
Education				0.284
Secondary	67 (20)	45 (14)	112 (34)	
Tertiary	85 (26)	63 (20)	148 (46)	
Missing system			64 (20)	
Professional category				0.745
Clinical staff	151 (47)	121 (37)	272 (84)	
Non-clinical staff	31 (10)	21 (6)	52 (16)	
Range of monthly salary^a^				0.135
< US$ 265	94 (29)	86 (27)	180 (56)	
US$ 265–688	81 (25)	47 (14)	128 (39)	
> US$ 688	3 (1)	0 (0)	3 (1)	
Missing system			13 (4)	
Marital status				0.046*
Married	67 (21)	73 (22)	140 (43)	
Not married	115 (35)	69 (21)	184 (57)	
Religion				0.209
Christian	175 (54)	136 (42)	311 (96)	
Non-Christian	6 (2)	7 (2)	13 (4)	

Source: WOTRO- COHEiSION Project Clinic Staff Survey (March-June, 2012)

*Pearson Chi-square test statistically significant at 0.05 level of significance

^a^GHC (Ghana Cedis) 2.1 is equivalent to US$ 1.0 (XE.com/currency converter, 13/08/2013)

^b^All percentages have been rounded to the nearest decimal point

### Health workers’ experiences with work conditions

The results showed that averagely, workers in urban health care facilities spent longer time traveling to work (mean = 33 min) than their counterparts in rural healthcare facilities (mean = 19 min), (*p* < 0.0001) (See Table 2). A greater percentage (89 %) of the workers was satisfied with their clinic’s physical environment; 11 % of them described their physical work conditions as disappointing. About 70 % of the staff interviewed expressed satisfaction with drug and resource availability (including water and electricity supply) in their workplaces; 28 % described the situation as disappointing; 21 % of staff in rural facilities expressed disappointment in drug and resource availability than their counterparts in urban health facilities (7 %, *p* = 0.0015).

**Table 2:** Comparison of work conditions and experiences of health staff in rural and urban health facilities (*n* = 324)

Work conditions	Geographical location			
	Rural (*n* = 182)	Urban (*n* = 142)	Total (*n* = 324)	*p*-value
	Mean (SD)	Mean (SD)	Mean (SD)	
Travel time to work in minutes on daily basis	19 (22)	33(32)	25(27)	0.0000*
Estimated extra work hours a day^a^	0.50(2.50)	0.50(2.20)	0.50(2.30)	0.9935
Number of minutes spent per patient at a time	13(11)	15(16)	14(13)	0.4250
Number of patients seen a day per staff	58(74)	44(40)	52(62)	0.0634**
Amount of allowance received a month for extra work done (in US$ equivalence)	45(68)	52(49)	48(61)	0.6783
Monthly financial income from part time work (in US$ equivalence)	162(164)	235(261)	210(230)	0.4569

Source: WOTRO- COHEiSION Project Clinic Staff Survey (March-June, 2012)

*SD* standard deviation

*Independent *t*-test of two-tail hypothesis is statistically significant at 0.05 level of significance

** Independent *t*-test of two-tail hypothesis is statistically significant at 0.10 level of significance

^a^Extra work hours calculated as the difference of actual hours spent at work a day and the expected work hours a day

Payment of financial incentives including monthly salaries was described by respondents as disappointing and perceived to be the least source of motivation by over 70 % of respondents; but 25 % of them described this incentive as satisfactory. Possibility for promotion and further education was an important source of motivation for 60 % staff interviewed (see Table 3).

**Table 3:** Rural–urban differences in staff motivation levels

	Rural	Urban	Total	*p*-value
Proxies for staff motivation	Freq. (%^a^)	Freq. (%)	Freq. (%)	
Physical work environment (*n* = 318)				0.2033
Disappointing	25(8 %)	11(3 %)	36(11 %)	
Satisfactory	154(49 %)	128(40 %)	282(89 %)	
Availability of resources and drugs (*n* = 321)				0.0015*
Disappointing	66(21 %)	24(7 %)	90(28 %)	
Satisfactory	113(35 %)	118(37 %)	231(72 %)	
Financial and extrinsic incentives (*n* = 312)				0.6216
Disappointing	131(42 %)	103(33 %)	234(75 %)	
Satisfactory	43(14 %)	35(11 %)	78(25 %)	
Job prospects and career development (*n* = 308)				0.1811
Disappointing	65(21 %)	58(19 %)	123(40 %)	
Satisfactory	110(36 %)	75(24 %)	185(60 %)	

Source: WOTRO- COHEiSION Project Clinic Staff Survey (March-June, 2012)

*Wilkoxon Mann–Whitney rank sum test statistically significant at 0.05 level of significance

^a^All percentages have been rounded up to the nearest decimal point

### Rural–urban differences in quality health care and patient safety standards

The results showed significant rural–urban differences in selected service output indicators such as number of deliveries per month and number of Human Immune Virus (HIV)/Acquired Immune Deficiency Syndrome (AIDS) preventive services per month in health facilities (*p* < 0.05) (See Table 4). Even though the staff numerical strengths in rural and urban facilities were not statistically different, on the average, facilities in rural areas conducted more deliveries in a month (mean = 17, SD = 18) than facilities in urban areas (mean = 7, SD = 13, *p* = 0.0112). Likewise, facilities in rural areas rendered more HIV/AIDS preventive services in a month (mean = 181, SD = 214) than facilities in urban areas (mean = 59, SD = 90, *p* = 0.0067) (See Table 4).

**Table 4:** Situational analysis of rural and urban health facilities (*n* = 64)

	Rural(*n* = 36)	Urban(*n* = 28)	
Factors	Mean(SD^b^)	Mean(SD)	*p*-value
Input indicators			
Staff strength per clinic	25(19)	24(25)	0.8670
Number of beds per clinic	11(10)	9(11)	0.4132
Process indicators			
Percentage of staff trained in health and safety in the last 12 months	39 % (48 %)	41 % (47 %)	0.8792
Number of orientation sessions by facility in the last 12 months	59(36)	49(43)	0.3067
Outputs indicators			
Number of deliveries in a month	17(18)	7(13)	0.0112*
Number of antenatal care (ANC) visits in a month	121(158)	77(125)	0.2269
Number of family planning (FP) services in a month	58(90)	59(152)	0.9619
Number of male condoms distributed in a month	96(193)	45(142)	0.2515
Number of preventive health services and screenings in a month^a^	52(19)	22(8)	0.1768
Number of chronic healthcare services in a month	125(171)	204(299)	0.1911
Number of HIV/AIDS preventive services in a month	181(214)	59(90)	0.0067*

Source: WOTRO- COHEiSION Project Clinic Staff Survey Data (March-June, 2012)

*Statistically significant at 0.05 level of significance using the independent *t*-test of two-tailed hypothesis

^a^These services include: Tuberculosis (TB), diabetes and cholesterol

^b^SD: Standard deviation

**Fig. 2:**
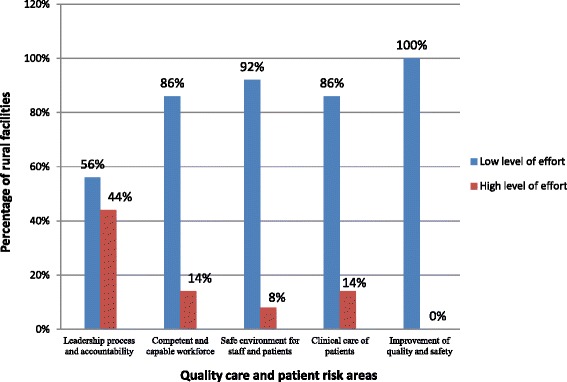
Levels of effort by rural health facilities towards quality health care and patient safety (*n* = 36)

**Fig. 3:**
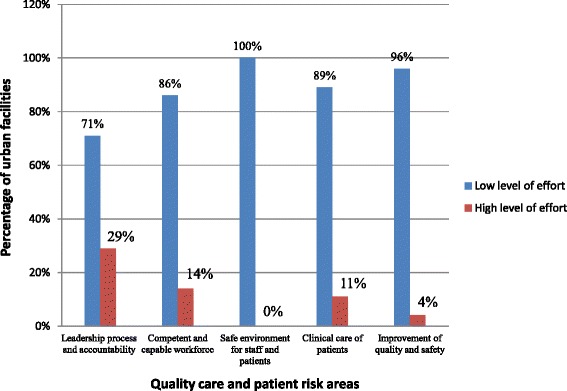
Levels of effort by urban health facilities towards quality health care and patient safety (*n* = 28)

As shown in Figs. 2 and 3, over 50 % of health facilities demonstrated low levels of effort towards risk reduction and patient safety. Quality care standards were particularly low in the areas of environmental safety for staff and patients, and quality improvement. Virtually all facilities (rural and urban) showed low levels of effort in these areas.

Most facilities surveyed performed better in leadership processes and accountability; workforce competency, and clinical care of patients. In comparative terms, more rural facilities had in place better standard practices in these areas than urban facilities. On the other hand, facilities in urban districts had in place better standard practices in the management and safe use of medications than facilities in rural areas (*p* = 0.0183). It was also found that facilities in rural areas had better protocols for staff training in resuscitation techniques than facilities in urban areas (*p* = 0.0219).

Ordered logistic regression was conducted to ascertain the relationship between facility geographical location and overall staff satisfaction levels and quality care health standards. “Overall staff satisfaction with work conditions” and “overall facility quality assessment score” were used as the dependent variables.

The independent variable of interest was the work location (rural/urban) of health staff. Control variables included in the regression model were: facility ownership (public/private), region (GAR/WR) and staff strength. Other variables controlled for were staff professional category (clinical/nonclinical), gender (male/female), education (tertiary/below tertiary) and monthly salary (<US$265/>US$265). Before these independent variables were included in the final regression model, multicollinearity diagnostics was conducted and none of them had a variance inflation factor (VIF) up to 10. The mean VIF was 1.43 which is adequate [11].

The ordered logistic regression showed that health facility location (rural/urban) has significant relationship with overall quality health care and patient safety standards. The results of this study found that overall staff motivation levels and quality health care in health facilities were not influenced by their geographical location (rural or urban). However, facility ownership (private/public) and numerical staff strengths had significant association with staff overall motivation levels and quality health care in health facilities (*p* < 0.05). Thus, the log odds of health workers in private facilities expressing greater satisfaction with work conditions was 2.9 times higher than workers in public facilities (*p* < 0.0001, CI = 1.9 – 4.5). Likewise, the log odds of private facilities exerting greater efforts towards quality care was 4.1 times greater than public facilities (*p* < 0.05, CI = 1.5 – 11.1). In addition, a unit increase in numerical strengths of staff increased the likelihood of improving staff motivation levels (OR = 1.0, *p* < 0.05, CI = 1.01 – 1.02) and overall quality health care performance of health facilities (OR = 1.1, *p* < 0.001, CI = 1.0 – 1.1) (See Table 5).

**Table 5:** Ordered logistic regression on determinants of staff motivation and quality health care in health facilities

	Dependent variables					
	Overall staff motivation score			Overall quality care score		
Independent variables	OR^a^	*p*-value	[95 % Conf. Interval]	OR	*p*-value	[95 % Conf. Interval]
Facility location						
Rural	0.07	0.767	(−0.37 0.50)	2.2	0.122	(0.8 5.8)
Urban	Ref	Ref	Ref	Ref	Ref	Ref
Facility ownership						
Private	2.9	0.0000*	(1.9 4.5)	4.1	0.005*	(1.5 11.1)
Public	Ref	Ref	Ref	Ref	Ref	Ref
Region						
GAR	0.86	0.510	(0.56 1.3)	0.69	0.432	(0.3 1.8)
WR	Ref	Ref	Ref	Ref	Ref	Ref
Clinic staff strength	1.0	0.001*	(1.01 1.02)	1.1	0.000*	(1.0 1.1)

Source: WOTRO- COHEiSION Project Clinic Staff Survey Data (March-June, 2012)

*Statistically significant at 0.05 level of significance

^a^OR = Odds Ratio

NOTE: Model 1: Overall staff satisfaction (Log Likelihood= -1002.765; Pseudo R2=0.0172; Prob > Chi2=0.0000)

NOTE: Model 2: Overall quality score (Log Likelihood= -185.956; Pseudo R2=0.0661; Prob > Chi2=0.0000)

## Discussion

Many countries in Africa including Ghana have not been able to attain all the health related MDGs before the end of 2015 [5], partly due to unequal distribution of health sector human resources in rural and urban areas. In Ghana, significant efforts have been made to bridge the widening rural–urban disparities in health sector resource allocation. The MoH, through the Human Resources for Health Development Directorate (HRHDD), has implemented several incentives to attract and retain essential health staff in rural and deprived areas [3]. Notwithstanding these interventions, staff motivation levels persistently remain low in these areas, thus raising concerns on the effectiveness of these interventions [6, 7].

This baseline quantitative study explored the work conditions of health staff and how these conditions incentivize or de-motivate them to render good quality health care to clients. It was found that generally quality health care, patient safety standards and staff motivation levels were not optimal. Work conditions were particularly perceived to be more de-motivating in rural areas, albeit quality health care standards were relatively better in rural than urban health facilities.

Major sources of de-motivation for health workers in rural areas were limited access to social amenities such as water and electricity, and regular stock out of essential drugs. These observations are consistent with findings of previous studies by Johnson et al. [7] and Lori et al. [10]. Perceived poor physical work environment and limited job prospects were the key demotivating factors for health staff working in rural facilities, similar to findings of previous studies in Ghana [7] and other countries [15–18].

The reviewed literature cited a number of reasons for the rural–urban imbalance in staff motivation levels including better opportunities for urban dwellers to pursue higher educational programmes alongside their jobs [7, 15–19]. These opportunities are virtually non-existent in rural areas where tertiary institutions and other professional development institutions are limited or absent.

Rural–urban differences were also found in staff satisfaction levels with financial incentives including monthly salaries and work allowances. Many health workers in rural facilities expressed disappointment in financial incentives than their counterparts in urban facilities. Previous studies [15–17, 19] have confirmed these geographical differences and concluded that rural health workers were less likely to express satisfaction with financial incentives because of the limited opportunities for part time work otherwise called moonlighting. Urban workers are likely to have multiple sources of income within a month while their rural counterparts might depend solely on their mainstream monthly salary.

The geographical imbalance in financial incentives for health workers, in the view of Stilwell et al. [16] and Dieleman et al. [17], does not only lead to concentration of skilled staff in better endowed urban areas, but also escalates into international workforce migration. An estimated cumulative number of 2,406 skilled health workers including medical officers, pharmacists, nurses, midwifes, medical laboratory technologists and radiologic technologists migrated from Ghana to Europe, United States of America and other developed countries between 1999 to 2003 in search of “greener pastures” [20]. According to the World Health Organization (WHO), the impact of this health professionals’ exodus on attainment of MDGs 4, 5 and 6 in developing countries is enormous and needs to be stemmed through collaborative efforts and effective staff motivation interventions [21].

In Ghana, about 70 % of physicians and professional nurses work in urban areas and the remaining 30 % work in rural areas where there is higher disease burden and demand for health care services [3]. This predicament is in contrast with Vietnam where an estimated 84 % of public sector health personnel work in rural areas where 80 % of the population lives [17].

Even though virtually no country in the world has been able to completely solve the rural–urban inequity in health sector human resource distribution [19], Thailand has made significant gains in this regard. Thailand in the 1990s started stemming down migration of rural health workers to urban areas through an implementation of wide range of strong financial incentives [22]. The Ministry of Health and Ghana Health Service need to learn from this approach to help close the wide rural–urban gap in health worker distribution and quality services delivery.

Use of financial incentives to motivate health sector workforce has been discussed in the literature with varying conclusions [7, 8, 17, 23, 24]. Some studies have argued that implementation of financial incentives without complementary non-financial incentives seldom improve health worker performance [7, 25]. Multifaceted staff motivation interventions have therefore been advocated.

In Ghana, the “knee-jerk” reaction to labour strikes and poor staff attitudes towards work is to increase monthly salaries. These interventions are often implemented without addressing equally important incentives such as transportation to work, career development plans and work organization. For instance, this study we found that travel time to work was averagely longer for urban dwellers (mean = 33 min) than rural dwellers (mean = 19 min), *p* < 0.0001. This finding could be due to the vehicular and human traffic situation in urban areas. On the other hand, workers in rural areas were more likely to stay closer to their health facilities and travel over shorter times to work compared to urban health workers [7].

Findings in this study suggest that interventions to improve work conditions should be tailored to the peculiar workplace constraints of staff than adopting “wholesale” interventions. Our study results show that experiences of staff differ based on geographical location of workplace. Perceived disincentives for staff in rural health facilities were basically inadequate financial remuneration, lack of career development opportunities and unavailability of social amenities. Urban health workers were more concerned with improvement of transportation system and other forms of non-financial incentives.

Apart from the rural–urban differences in motivation levels of staff, the study also explored the quality situation in surveyed health facilities. The study found statistically significant rural–urban differences in health facility efficiency in delivery of primary health care services. Rural facilities averagely conducted more deliveries in a month (mean = 39, SD = 18) than urban facilities (mean = 7, SD = 13), *p* < 0.05. Likewise, rural facilities provided more HIV/AIDS preventive services in a month (mean = 181, SD = 214) than urban facilities (mean = 59, SD = 90), *p* < 0.05).

The outcome of this situational analysis is consistent with the primary health care concept in Ghana, where rural and peri-urban health facilities are expected to render mainly basic health care services. Morever, many urban health facilities are private-for-profit and would likely render more curative/chronic services than primary health care services for profit purposes.

In addition, the levels of efforts towards risk reduction and quality improvement in rural and urban facilities were generally low. While rural facilities performed minimally in quality improvement, urban facilities were more deficient in environmental safety for staff and patients.

Standard practices in leadership processes and accountability were relatively adequate in all 64 surveyed facilities with rural facilities performing better than urban facilities. In addition, most rural facilities had evidence of training their clinical care staff in resuscitative techniques than urban facilities (*p* = 0.0219). On the other hand, many urban facilities were found to adhere better to protocols on safe use of medications than rural facilities (*p* = 0.0183).

These findings could be explained by the relatively dominant Faith-based Organization (FBO) clinics in rural areas in Ghana. These FBO clinics (categorized under private-not-for-profit) are known to maintain better health care quality standards compared to other categories of facilities [26–28].

Ordered logistic regression was further conducted to ascertain the relationship between facility geographical location (rural/urban) and staff motivation levels and medical technical quality.

Overall, the results showed that geographical location of health facilities has no association with quality health care performance of health facilities and staff motivation levels. Facility ownership and staff strength were however found to have association with quality health care and staff motivation levels.

These observations could be attributed to the cadre of health facilities sampled for the study. Clinics and health centres under Ghana’s healthcare system often render primary healthcare services using similar cadre of health professionals in terms of qualification and training. Likewise, remunerations especially in public facilities usually do not vary in rural and urban facilities. These realities perhaps explain why facility ownership and staff numerical strengths, appeared to be stronger predictors of staff motivation levels and overall quality health care performance of health facilities.

Many previous studies concluded that quality health care in general was better in urban than rural facilities [26–28], however this study revealed that, in terms of efficiency, facilities in rural areas were more efficient in rendering preventive primary healthcare services than urban facilities.

Furthermore, since overall quality health care and patient safety standards were low in rural and urban accredited health facilities, the NHIA accreditation unit in collaboration with the GHS quality assurance unit should intensify regular post accreditation monitoring to ensure that facilities adhere to quality care standards after accreditation. The current post monitoring protocols using district mutual health insurance schemes and claims vetting do not seem to help maintain quality health care standards in accredited facilities [29].

Moreover, the ministry of health through the HRHDD should ensure that existing policies on rural health worker motivation are effectively implemented alongside monitoring systems to ensure the true beneficiaries of these motivational packages get them. Infrastructural improvement, facility resourcing, and career development plans are important areas the MoH should prioritize as retention strategies for health workers especially in rural areas. Likewise, supporting staff with transportation to work and accommodation should be considered for workers in urban areas since these are their major sources of de-motivation at work.

## Limitations

There are some limitations associated with this study that must be acknowledged. First, the study was conducted among workers of primary healthcare facilities which have different work arrangements and conditions. It is possible responses of higher level facilities (e.g., hospitals) will differ. Secondly, the study was conducted in two out of ten regions in Ghana and the findings might not necessarily reflect the working conditions of health workers in other regions. In view of these limitations, the results should be generalized with caution.

Future researchers should consider expanding the sample size to include more regions and health care facilities across the northern and southern parts of Ghana. Comparing staff experiences and quality health care situations in NHIS accredited and non-accredited facilities could also be considered in future studies to determine whether or not accreditation status of rural and urban health care facilities has a relationship with staff motivation and quality health care performance.

## Conclusions

Geographic location of health care facilities has an association with their efficiency levels in the delivery of health services to clients. However, quality health care standards and staff motivation levels were not significantly different rural and urban facilities, perhaps due to their homogeneous nature in this study.

Nonetheless, it was found that, many health workers in rural health facilities expressed disappointment in availability of resources and drugs than those in urban health facilities. Staff motivation and quality improvement interventions should therefore be guided by these dynamics to ensure effective implementation.

For Ghana to meet the new sustainable development goals, there is the need to address the existing rural-urban disparities in health worker motivation and health care quality standards Addressing rural-urban inequities in human and material resources will help control rural-urban migration of health workforce and reduce the concentration of skilled staff in better endowed urban areas.

## Abbreviations

COHSASA, council for health services accreditation of southern Africa; ERC, ethical review committee; FBOs, Faith-based organizations; FGDs, Focused group discussions; GAR, Greater Accra region; GDP, gross domestic product; GHS, Ghana health service; GHWO, Ghana health workers observatory; GoG, Government of Ghana; HRHDD, human resource for health development directorate; IFAD, International fund for agricultural development; JCI, joint commission international; MDAs, ministries departments and agencies; MDGs, millennium development goals; MoH, Ministry of health; NHIA, National health insurance authority; NHIL, National health insurance levy; NHIS, National health insurance scheme; PCA, principal component analysis; PDA, pocket digital assistant; PHC, population and housing census; TBAs, traditional birth attendance; VIF, variance inflation factor; WHO, World health organization; WR, Western region

## Footnotes

1 COHEiSION Project is a four year (2011 to 2015) randomized control trail study on the topic: “Towards client-centred health insurance system in Ghana”. The project is a collaborative effort of three universities in the Netherlands (Amsterdam Institute of Global Health and Development (AIGHD), University of Amsterdam; University of Groningen and Vrije University of Amsterdam) and the Noguchi Memorial Institute for Medical Research, University of Ghana, Legon. The project is being conducted in the Western and Greater Accra regions from client, health provider and insurer perspectives, each focused on by a PhD student. Other local stakeholders of the project are the Ghana Health Service (GHS), Ministry of Health (MoH), National Health Insurance Authority (NHIA), Society of Private Medical and Dental Practitioners (SPMDP) and the Christian Health Association of Ghana (CHAG)

2 According to the GSS/PHC report, rural areas are geographical locations with less than 5,000 persons

3 According to the GSS/PHC report, urban areas are geographical locations with more than 5,000 persons

4 Clinical staff as used in this context include: doctors, medical assistants, nurses, midwives, pharmacists, laboratory personnel, nurse-assistants and community health nurses. Non-clinical staff on the other hand include: administrators, NHIS contact persons, receptionists, accountants and laborers.
